# Off-label use of drug-coated balloon (Optilume™) for urethral stricture in a 16-year-old boy: the FIRST pediatric case report

**DOI:** 10.3389/fped.2025.1737866

**Published:** 2026-01-15

**Authors:** Klaudia Korlacka, Piotr Bryniarski

**Affiliations:** Department of Urology in Zabrze, Medical University of Silesia in Katowice, Katowice, Poland

**Keywords:** adverse effects of cardiac surgery, endourological surgery, paclitaxel, urethral stricture in children, urethral dilation, urethral stricture

## Abstract

Urethral strictures that are not related to urological procedures or severe urethral trauma are rare in the pediatric population. Potential etiological factors include bladder catheterization during advanced surgical procedures and in intensive care units. We present the first reported off-label, minimally invasive treatment of a long-segment anterior urethral stricture in a teenager following cardiac surgery, using a paclitaxel-coated balloon (Optilume™). During the diagnosis of urethral stricture, cystoscopy and urethral biopsy were performed, which ruled out balanitis xerotica obliterans on histopathological examination. After mechanical dilation of the urethra to 18Fr over a period of two months, restenosis was observed, confirmed by uroflowmetry, with a *Q*_max_ of 4.6 mL/s and a voided volume of 202.9 mL. An 18Fr (6 mm) balloon with a length of 50 mm was used during the treatment. The procedure was performed under fluoroscopic guidance, without the use of a cystoscope sheath, using a 0.038″ OTW guidewire. The balloon was inflated to a pressure of 10 atm for 5 min. After the procedure, a 14Fr Foley catheter was left in place for 72 h. The perioperative course was uneventful, with no local or systemic reactions. Uroflowmetry performed 72 h after the procedure showed a *Q*_max_ of 20.7 mL/s with a voided volume of 283.5 mL. At the 3-month follow-up, *Q*_max_ was 19.6 mL/s with a voided volume of 219.7 mL, and at 8 months follow-up, *Q*_max_ was 18.4 mL/s with a voided volume of 160.5 mL, without post-void residual urine. There are currently no published studies in the pediatric literature evaluating treatment with drug-coated balloons (DCBs). According to the EAU/ESPU guidelines, there are no dedicated recommendations for the management of urethral strictures in children, as this condition is rare in the pediatric population and requires individualized treatment, especially in children with significant comorbidities. Optilume™ may represent a potential safe, minimally invasive therapeutic option to consider only in exceptional, carefully selected pediatric cases. However, this off-label use requires careful counseling, and long-term follow-up to assess the durability and late safety outcomes.

## Introduction

Urethral strictures (US) that are not caused by urinary tract anomalies in the pediatric population are rare urological conditions (1). Their prevalence is estimated at <1%, and they most commonly occur in boys over 10 years of age (2, 3). The main causes of US include trauma (especially perineal injury), postoperative complications (e.g., after hypospadias repair), and iatrogenic changes resulting from endoscopic procedures, as well as prolonged intraoperative and postoperative catheterization (2, 3). Even short-term catheterization under compromised tissue perfusion can lead to the development of urethral strictures.

An important cause of secondary urethral strictures in boys and adolescents is lichen sclerosus (BXO—balanitis xerotica obliterans), a chronic inflammatory dermatosis of unknown etiology (4). In cases of urethral involvement, chronic inflammation and secondary fibrosis within the mucosa may affect both the external meatus and the bulbar urethra, which can lead to long-segment strictures that are resistant to treatment.

Urethral strictures in adolescents are a significant clinical problem and can lead to a marked deterioration in quality of life. They typically manifest as a weak urinary stream, post-void residual urine, urinary tract infections, increased voiding frequency, and secondary detrusor hypertrophy with voiding dysfunction. Treatment of long-segment strictures (>1 cm) using traditional methods such as meatotomy, internal urethrotomy, and urethral dilatation is associated with a high recurrence rate. End-to-end urethroplasty and reconstructive procedures using buccal mucosal grafts (BMG) or inner foreskin, including single- and two-stage repairs, are typically required in cases of recurrence and confirmed inflammatory-fibrotic changes.

In the present case, the patient's severe cardiac history and chronic anticoagulation significantly increased the anesthetic and perioperative bleeding risks associated with open urethral reconstruction. Given the early recurrence after staged mechanical dilations and the lack of pediatric evidence for drug-coated balloons, we pursued an individualized, transparent off-label approach with Optilume™ as a minimally invasive, proof-of-concept option, coupled with structured functional follow-up and planned long-term endoscopic surveillance.

In recent years, new endoscopic treatment techniques have emerged involving balloons coated with antiproliferative drugs. The Optilume™ balloon, coated with paclitaxel, has shown positive results in clinical trials in adult men with recurrent urethral strictures and was approved for clinical use by the FDA in December 2021 (5–8). Paclitaxel, a cytostatic drug from the taxane group, inhibits re-scarring by limiting smooth muscle cell and fibroblast proliferation. When used in drug-coated balloon systems, it acts locally at the site of injury.

There are currently no published pediatric studies evaluating treatment with drug-coated balloons (DCBs) (1). Likewise, there are no dedicated EAU/ESPU recommendations for the treatment of urethral strictures in children, as these conditions are rare, typically involve small and heterogeneous patient groups, and require a personalized treatment strategy, especially in children with comorbidities (e.g., after cardiac surgery, with BXO, after hypospadias repair).

The aim of this report is to present the first successful, minimally invasive treatment using the Optilume™ drug-coated balloon in a 16-year-old boy with a long-segment anterior urethral stricture.

## Case description

### Clinical course

**Figure 1:**
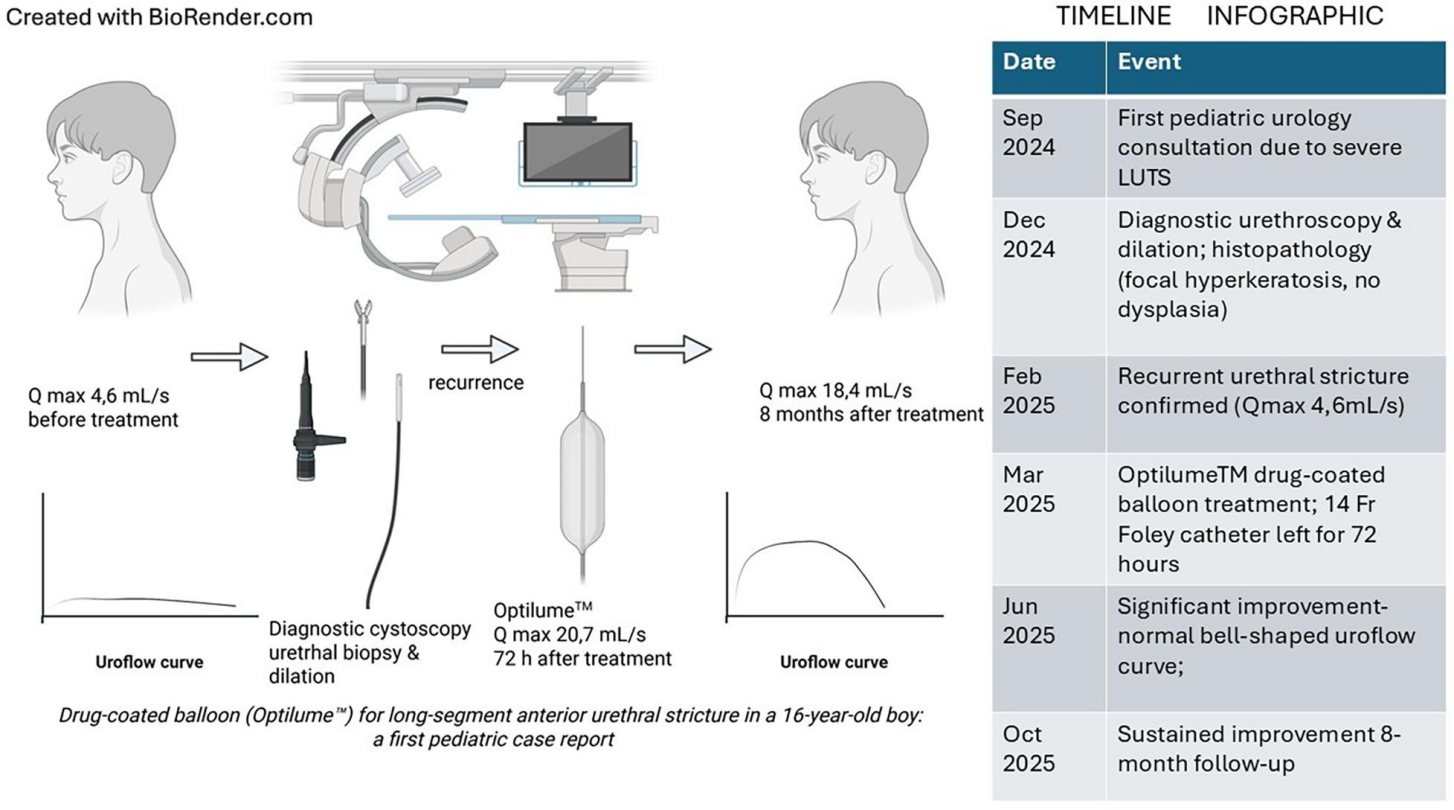
Timeline of the clinical course of a 16-year-old boy with long-segment anterior urethral stricture treated with a paclitaxel-coated balloon (Optilume™). The infographic summarizes the main diagnostic and therapeutic milestones, diagnostic urethroscopy with biopsy, recurrence of the stricture, drug-coated balloon treatment, and follow-up results confirming sustained improvement in uroflowmetry and symptom resolution at 8 months.

The patient's first urological consultation took place at the age of 15. He was referred due to severe lower urinary tract symptoms (LUTS)—frequent daytime and nighttime urination accompanied by urgency, incomplete bladder emptying, and occasional urinary incontinence, without evidence of urinary tract infection. Physical examination revealed normally developed external genitalia, with no phimosis, no features of BXO, and no narrowing of the external urethral meatus. Ultrasound showed a smooth-walled bladder with significant post-void residual urine (80–100 mL). An attempt at bladder catheterization with an 8Fr catheter was unsuccessful at the level of the anterior urethra, just behind the fossa navicularis. The patient had a history of congenital heart disease in the form of an atrioventricular septal defect (AVSD) with mitral valve regurgitation and atrioventricular conduction disturbances (second-degree AV block). He underwent cardiac surgery with cardiopulmonary bypass at the age of 14, during which a prosthetic valve was implanted. He remains under cardiological follow-up, treated with lisinopril, spironolactone, and warfarin. The clinical timeline, from the onset of symptoms to 8 months of follow-up after treatment with Optilume™, is shown in Figure 1.

### Diagnostic assessment

At the age of 15, diagnostic urethroscopy under general anesthesia confirmed a long-segment (∼3 cm) narrowing of the anterior urethra, beginning 7 mm distal to the external meatus and involving the fossa navicularis region. The stricture was treated with a single dilation to 18Fr, and a biopsy was obtained. Histopathology demonstrated stratified squamous epithelium with focal hyperkeratosis, without dysplasia. A 16Fr Foley catheter was left for 7 days. Despite recommendations, the patient refused self-dilatation. Within two months, restenosis occurred and two experienced urologists were unable to reinsert the catheter. Follow-up uroflowmetry showed *Q*_max_ 4.6 mL/s, voided volume 202.9 mL, flat flow curve, Qave 4 mL/s, flow time 50.7 s, time to *Q*_max_ 30.5 s, and 2.2 mL/s at 2 s of flow.

### Therapeutic intervention

At the age of 16 (when the patient could provide informed consent), he was qualified for treatment with a paclitaxel-coated balloon (Optilume™). The procedure was performed under general anesthesia and fluoroscopic guidance, without a cystoscope sheath, using a 0.038″ OTW guidewire. An 18Fr (6 mm) × 50 mm drug-coated balloon was used. The 18Fr (6 mm) diameter was selected as the smallest available Optilume™ size to minimize the risk of over-dilation in an andolescent urethra; the 50 mm working length was chosen to fully cover the stricture with adequate proximal and distal marings (0,5–1 cm). Balloon handling and preparation followed the manufacturer's instructions for use: the device was kept within its protective sheath until insertion, and the drug-coated balloon was not immersed in saline and was not wiped prior to urethral insertion to avoid disruption the coating; the inflation device was primed with sterile saline with air removed from the system. After positioning across the stricture the balloon was kept uninflated *in situ* for ≥60 s to allow coating hydration before inflation.

**Figure 2:**
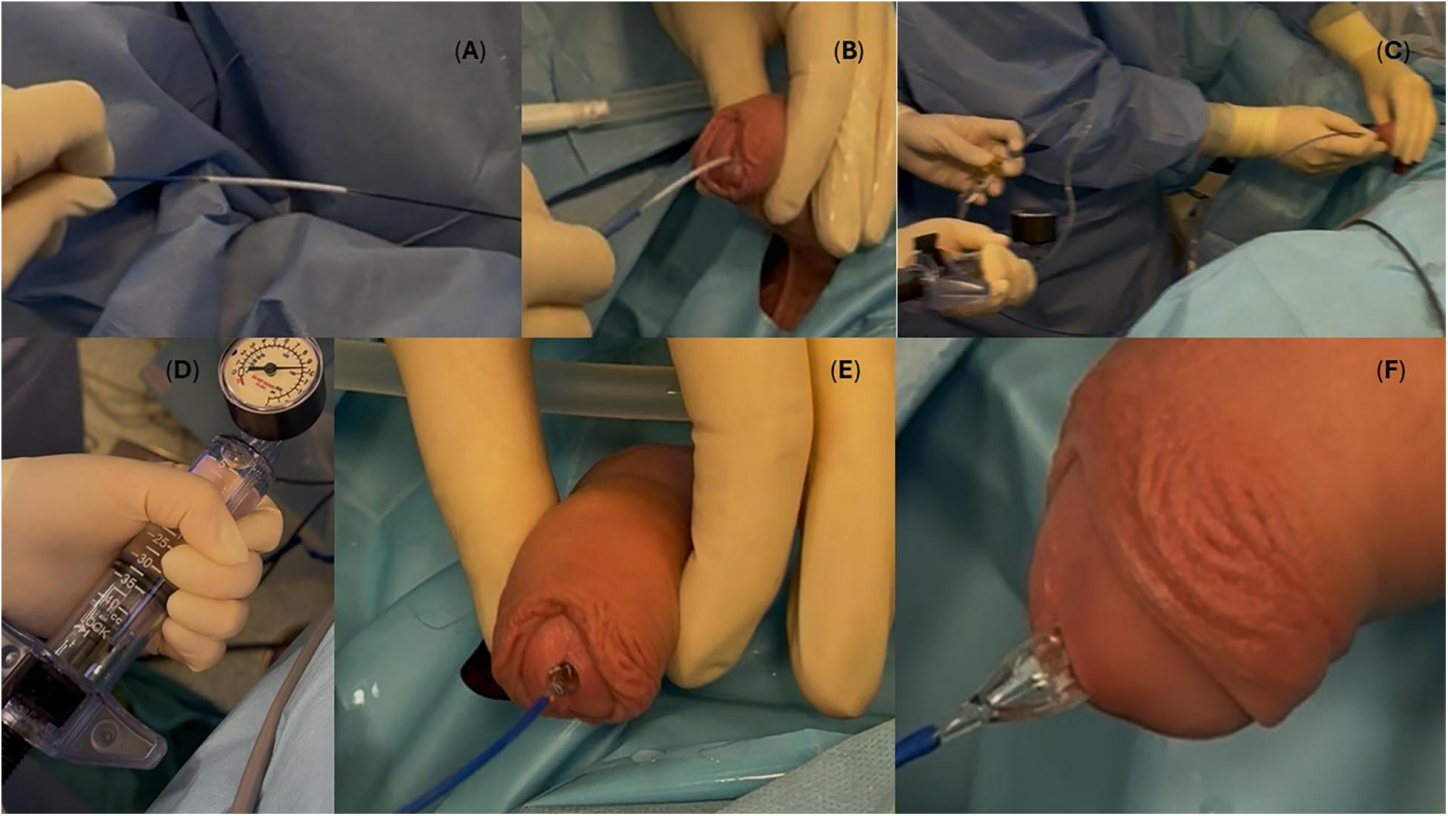
Intraoperative stages of the Optilume™ drug-coated balloon procedure in a 16-year-old boy with a long-segment anterior urethral stricture. **(A)** Introduction of a 0.038″ guidewire under fluoroscopic control. **(B)** Passage of the drug-coated balloon over the guidewire to the stricture site. **(C)** Fluoroscopic verification of balloon position. **(D–F)** Balloon inflation to 10 atm for 5 min.

The balloon was inflated to 10 atm for 5 min (Figure 2). After the procedure, a 14Fr Foley catheter was left for 72 h. The perioperative course was uneventful, with no local or systemic adverse events and stable coagulation parameters. Perioperative antibiotic prophylaxis consisted of gentamicin administered intravenously 30 min before the procedurę and thromboprophylaxis was provided with enoxaparin 0,4 mL subcutaneously once daily.

### Follow-up and outcomes

Uroflowmetry performed 72 h after catheter removal showed a bell-shaped flow curve with marked improvement in voiding parameters (Table 1). Since the procedure (March 2025), the patient has remained asymptomatic, performs clean intermittent catheterization (CIC) using a 14Fr hydrophilic catheter (Coloplast), and reports no LUTS. At 8-month follow-up, uroflowmetry demonstrated sustained improvement (Table 1), with no post-void residual urine. Systematic surveillance for potential paclitaxel-related adverse effects included clinical assessment for local urethral inflammation, hematuria, dysuria, urinary tract infection, hypersensitivity reactions and systemic symptoms; no such events were observed during short-term follow-up. Planned follow-up includes endoscopic evaluation at 12 months and uroflowmetry every 3–4 months to assess long-term durability of the treatment effect.

**Table 1:** Comparison of uroflowmetry parameters before and after Optilume™ treatment.

Parameter	Before Optilume™	72 h after Optilume™	15 weeks after Optilume™	32 weeks after Optilume™
*Q*_max_ (mL/s)	4.6	20.7	19.6	18.4
*Q*_avg_ (mL/s)	4.0	13.6	13.0	12.8
Voided volume (mL)	202.9	283.5	219.7	160.5
Flow time (s)	50.7	20.8	16.8	12.5
Time to *Q*_max_ (s)	30.5	8.6	9.5	6.9
Flow rate at 2 s (mL/s)	2.2	10.0	10.3	11.4

## Discussion

Urethral strictures in pediatric patients after cardiac surgery are rarely reported in postoperative outcome analyses. Their incidence may appear low due to routine use of silicone catheters or underdiagnosis when attention is focused on perioperative survival. In a 1985 study by Prabhu et al. involving 221 children undergoing cardiac surgery, 10 experienced voiding difficulties after catheter removal, and 6 of them (2.7%) were diagnosed with symptomatic urethral strictures requiring treatment (9). Development of strictures may be associated with perioperative perineal ischemia resulting from reduced perfusion pressure, prolonged catheterization, and secondary urinary tract infections. These strictures usually involve the anterior urethra and may present weeks after surgery, posing a diagnostic challenge. Our patient did not initially associate his symptoms with previous cardiac surgery and reported no prior urinary complaints. Considering his age, cosmetic concerns, and the risk of recurrent scarring, open surgical repair was regarded as a last resort. His strong preference to avoid urethroplasty also influenced shared decision-making. Importantly, surgery in a patient with complex congenital heart disease and a prosthetic mitral valve, requiring chronic anticoagulation, carries increased risk of perioperative bleeding complications. Paclitaxel has already been extensively studied and safely used as an antimitotic drug in cardiovascular interventions (5).

Some surgeons prefer direct vision internal urethrotomy (DVIU) due to direct visualization during treatment. According to EAU Guidelines, there is no clear evidence that dilation is superior to DVIU (or vice versa), and therefore indications overlap (10). However, several reports suggest that balloon dilation may provide improved long-term outcomes compared with mechanical dilation or DVIU (10–12). According to Vetterlein et al., who analyzed 22 studies including 682 pediatric patients, the cumulative success rate of endoscopic treatment (DVIU/dilation) vs. urethroplasty was 46% vs. 84%, respectively. They recommend open surgical reconstruction in order to avoid repeated interventions (1). In our opinion, preliminary urethral calibration with urethroscopy may improve patient selection for drug-coated balloon (DCB) treatment in children.

Despite evidence from the ROBUST I–III clinical trials confirming the efficacy of the Optilume™ paclitaxel-coated balloon in adult patients with recurrent anterior strictures, no pediatric studies are currently available (5, 6, 8). Importantly, evidence supporting Optilume™ derives from adult trials and should not be extrapolated to pediatric populations as equivalence. In children and adolescents, urethral caliber, etiology, tissue healing, and long-term exposure considerations differ, and there are no standardized pediatric protocols or pharmacovigilance frameworks for drug-eluting urethral devices. Therefore, comparisons with the ROBUST study results are presented only as contextual data for adults, not as evidence of efficacy in minors (Table 2). This clearly demonstrates the need for prospective pediatric trials. The clinical trial criteria included recurrent strictures ≤2–3 cm in the anterior/hanging urethra after failed endoscopy, while exclusion criteria involved pediatric patients, prior radiotherapy, BXO, and severe strictures requiring open surgery. Our patient had: rapid recurrence after dilation, no BXO on histopathology, and a long-segment anterior stricture, which made him suitable for off-label Optilume™ therapy after informed consent from the patient and family; consent from the bioethics committee was not required.

**Table 2:** Comparison of results from ROBUST I–III clinical trials (Optilume™).

Parameter	ROBUST I—5-Year Outcomes NCT03014726	ROBUST II (after 6 months and 1 year) NCT03270384	ROBUST III (after 2 years) NCT03499964
Population	53 patients	16 patients	79 patients
Mean *Q*_max_	19.9 mL/s (*p* < 0.01)	20.8 mL/s (*p* < 0.01)	12.6 mL/s (*p* = 0.003)
Therapeutic success	58% (functional success)	73% (anatomic success)	78.5% (functional success)
Durability of effect	Sustained for 5 years	1-Year efficacy	Durable at 2 years
Complications	No serious AEs	No serious AEs	No serious AEs

Balloon diameter may influence clinical success. In ROBUST III, most adults were treated with a 30Fr balloon (5). In adolescents, pre-procedural urethrography is essential to ensure adequate balloon coverage (0.5–1 cm proximal and distal to the stricture). We believe that the smallest Optilume™ balloon size (18Fr/6 mm) is appropriate for adolescent patients and enables clinically reliable improvements in uroflowmetry.

The data obtained are preliminary. The limitation of the case report reflects a single case and provides only short-term follow-up (8 months), which precludes conclusions regarding long-term durability and late safety. Additionally, pediatric-specific pharmacokinetic and exposure data for local paclitaxel delivery in the urethra are unavailable. A structured 36-month follow-up is planned, including uroflowmetry every 3–4 months and cystoscopic evaluation at 12, 24, and 36 months, to assess recurrence, late adverse events, and the need for further interventions.

Given the rarity and heterogeneity of pediatric urethral strictures, future evidence generation should prioritize multicenter registries and standardized pediatric case series capturing anatomy, perioperative protocols, functional outcomes, and long-term safety endpoints, including dedicated pharmacovigilance reporting for drug-eluting devices.

## Conclusions

The use of a paclitaxel-coated balloon (Optilume™) in a carefully selected adolescent patient was feasible and associated with marked short-term functional improvement in short-term follow-up. No local or systemic adverse events were observed through 8 months. Complete restoration of urethral patency, confirmed by uroflowmetry, and full resolution of LUTS were achieved without the need for open surgical reconstruction. To our knowledge, this is the first published report describing the use of the Optilume™ balloon in a pediatric patient. Extended follow-up will be required to assess long-term durability in adolescents. At present, this technique cannot be recommended for routine pediatric practice and should be limited to exceptional, carefully selected cases with transparent intitutional oversight and long-term follow-up.

Multicenter prospective studies are needed to evaluate DCB treatment in urethral strictures among adolescents, especially those with high disease burden. Early uroflowmetry screening may be advisable in patients at risk, such as those following cardiac surgery or undergoing prolonged bladder catheterization.

## Patient perspective

The adolescent patient stated that he would choose the same treatment again, and if symptoms recur, he is highly motivated to undergo repeat endoscopic management to avoid open urethral reconstruction.

## Data Availability

The datasets presented in this article are not readily available because of ethical and privacy restrictions. Requests to access the datasets should be directed to the corresponding author.
